# Navigating the red: Diagnostic dilemma of erythema and diffuse body rash post- intrathecal baclofen pump implantation

**DOI:** 10.1016/j.inpm.2025.100584

**Published:** 2025-04-15

**Authors:** Chelsey Hoffmann, Annie Howrigon, Jennifer Hollister, William D. Mauck, Oludare O. Olatoye

**Affiliations:** Department of Anesthesiology and Perioperative Medicine, Division of Pain Medicine, Mayo Clinic, Rochester, MN, 55905, USA

**Keywords:** Pain pump, Intrathecal drug delivery device, Infection, Dermatology

## Abstract

Intrathecal drug delivery system (IDDS) infections can be localized to the pump pocket site and/or the catheter insertion site or become systemic, all potentially resulting in IDDS explant. Given the well-established effectiveness of IDDS for chronic non-cancer pain, cancer-associated pain, and spasticity, clinicians must differentiate between localized and systemic post-operative IDDS infections, as well as identify other causes of post-surgical skin irritation while avoiding unnecessary device explanation and therapy interruption.

In this letter-to-the-editor, we describe and discuss the importance of taking a thorough patient history and utilizing both critical clinical decision-making as well as expertise from other subspecialists to care for IDDS patients and navigate problematic skin reactions following implant.

**Dear Editor,**

Intrathecal drug delivery systems (IDDS) have been found to be an economically effective treatment, for appropriately selected patients, suffering from chronic non-cancer pain, cancer-associated pain, and spasticity [1,2]. IDDS involves a reservoir pump which is filled with medication and connected to a catheter, delivering medication into the intrathecal space. The entire system is implanted subcutaneously with the pump often being placed on either side of the abdomen in what is known as a pump “pocket,” though it can be placed in other areas such as the gluteal region or thigh depending upon the patient's body habitus and tissue distribution. The pump is connected to a catheter which is subcutaneously tunneled to the spine and placed into the intrathecal space.

Risks of IDDS implantation include bleeding, infection, injury to surrounding structures, cerebrospinal fluid leaks, hardware malfunction (i.e., pump or catheter), and intrathecal granuloma formation [3]. IDDS infections can range from a localized pump pocket or catheter site infection to a systemic infection and may require IDDS explanation. Guidelines on the prevention of IDDS surgical site infections exist from the Polyanalgesic Consensus Conference (PACC) and continue to be routinely updated as new evidence emerges to support best practices [1,2].

Differentiating between localized pump pocket infections and other causes of cutaneous irritation can be challenging for clinicians. Previously published case reports have identified various causes for skin reactions near IDDS pump sites including contact dermatitis, hypersensitivity reactions such as medication or hardware metal allergic reaction, or mechanical friction from materials or objects rubbing against the area [[4], [5], [6]].

We cared for a 74-year-old gentleman diagnosed with Amyotrophic Lateral Sclerosis (ALS) who had associated bilateral lower extremity spasticity. He was referred to interventional pain medicine for consideration of IDDS baclofen implant for management of medically intractable lower extremity spasticity secondary to his ALS following a successful intrathecal Baclofen test dose of 50 mcg with improved spasticity and ease of wheelchair functional transfer.

The patient underwent an uncomplicated and straightforward IDDS baclofen implant in November 2024 with entry at the L3-4 level and advancement of the intrathecal catheter under live fluoroscopy to the level of T8. A Medtronic 40 cc pump was implanted in the lower right quadrant of the abdomen, infusing Baclofen 500 mcg/mL at a dose of 100 mcg/day via continuous basal infusion. Two grams of intravenous cefazolin was administered intraoperatively for surgical infection prophylaxis. He was admitted to the hospital overnight for observation, as is standard practice, and subsequently discharged to a skilled nursing facility with well approximated incisions and no notable concerns (Fig. 1). Given his wheelchair dependency with a presumed higher infection risk, he was prescribed a seven-day course of cefadroxil 500 mg twice daily for surgical infection prophylaxis.

**Fig. 1:**
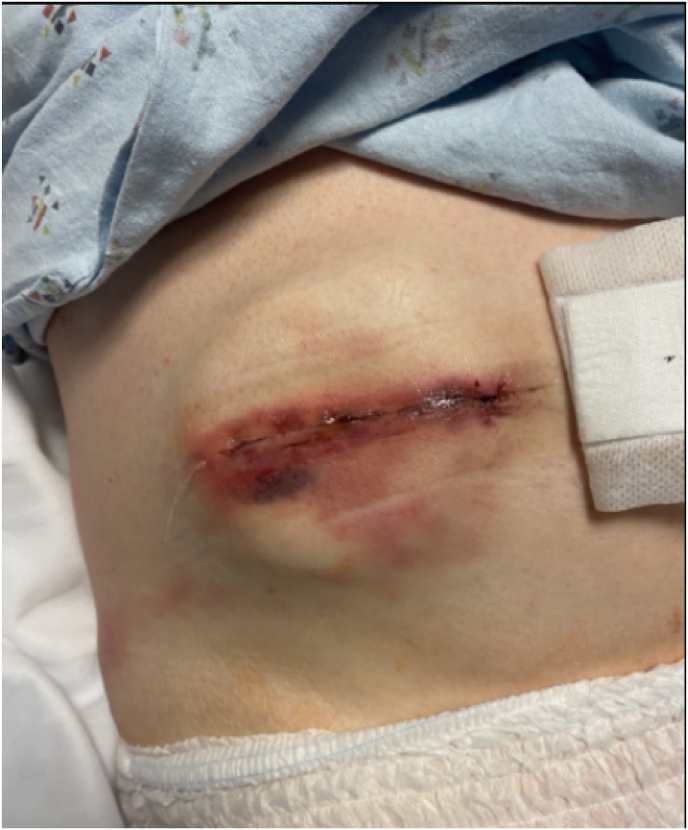
Pump reservoir incisional site on POD 1∗ ∗Note: photo was taken with patient in the supine position in his hospital bed.

Post-discharge, there were no reported concerns until post-operative day (POD 37) when mild erythema, a few raised non-pruritic raised and granulation tissue were noted around the pump reservoir site during an evaluation at the patient's skilled nursing facility (Fig. 2). The patient self-administered topical triamcinolone, dilute bleach, and ciclopirox to the affected area, believing he had tinea corporis based on his previous history with the condition. He was subsequently referred to the pain clinic for further evaluation and was advised to refrain from topical application of triamcinolone and dilute bleach which are known cutaneous irritants. Topical Aquaphor was recommended for itchiness. A subsequent evaluation about one week later (POD 47) showed worsened erythema around the pump reservoir site along with an increase in raised bumps on the incision (Fig. 3). The patient continued to deny any systemic symptoms. However, given the appearance of the reservoir site and growing concerns for superficial infection, a seven-day course of oral Bactrim was prescribed.

**Fig. 2:**
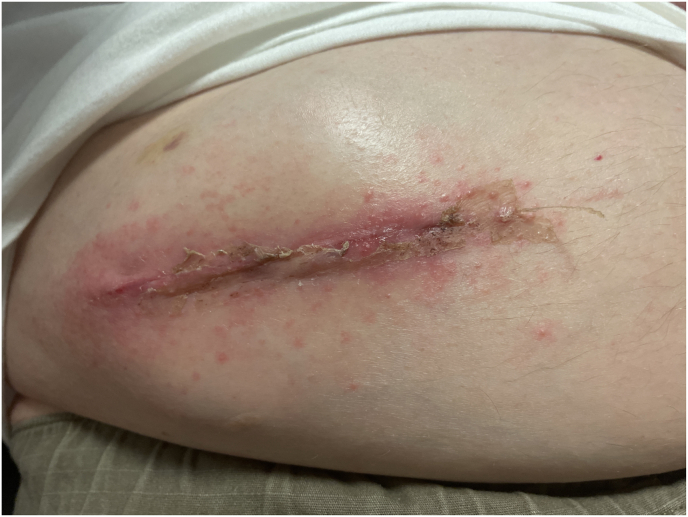
Pump reservoir incision site on POD 37∗ ∗Note: photo taken while patient seated in his electric wheelchair.

**Fig. 3:**
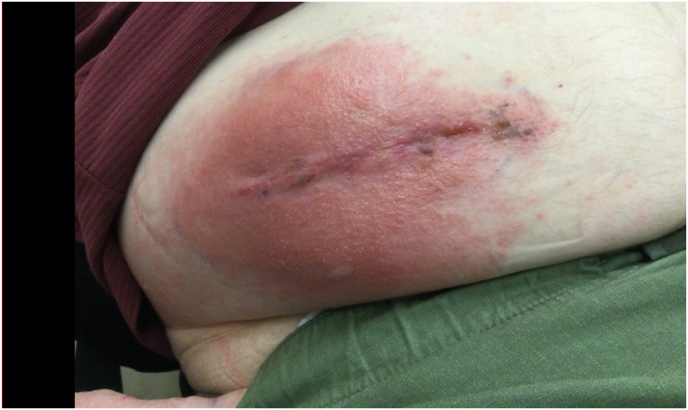
Pump reservoir incision site on POD 47.

A return visit on (POD 51) showed worsened erythema, mild swelling around the pump reservoir site and satellite lesion involving the entire chest wall anteriorly and posteriorly. At this point, infectious disease was contacted. They recommended a switch from oral Bactrim to oral Augmentin and doxycycline for 7 days. Device explant was also strongly recommended for consideration if skin changes persisted despite escalation of antibiotic therapy. On (POD 54), erythema and satellite lesions appeared to have worsened (Fig. 4). Prior to scheduling a device explant, a referral was placed to Dermatology given the nature and odd spread of the rash involving the back, and proximal lower extremity. The patient was subsequently seen in-person by Dermatology, and it was discovered that the patient was utilizing Cardinal Health wipes for body cleansing at his skilled nursing facility which was different from the body wipes he had previously used. The use of these wipes had not been previously disclosed to the pain clinic. Cardinal Health wipes contain Methylchloroisothiazolinone/methylisothiazolinone (MCI/MI) which is a known contact allergen [7]. He was additionally exposed to Aquaphor which contains lanolin – another potential contact allergen. Dermatology suspected “body wipe induced contact dermatitis” and recommended that the patient utilize Tacrolimus ointment twice daily to the affected areas as well as CeraVe Anti-Itch cream (over the counter) twice daily. They also recommended the following oral prednisone taper: 50 mg for two days, 40 mg for two days, 30 for two days, 20 mg for two days, 10 mg for two days, and 5 mg for two days. With the recommendations from dermatology, the patient's skin lesions significantly improved along with resolution of the erythema around the incision site (Fig. 5). The patient was seen for weekly follow-up appointments thereafter, with the pump site continuing to show resolution of the prior superficial skin rash.

**Fig. 4:**
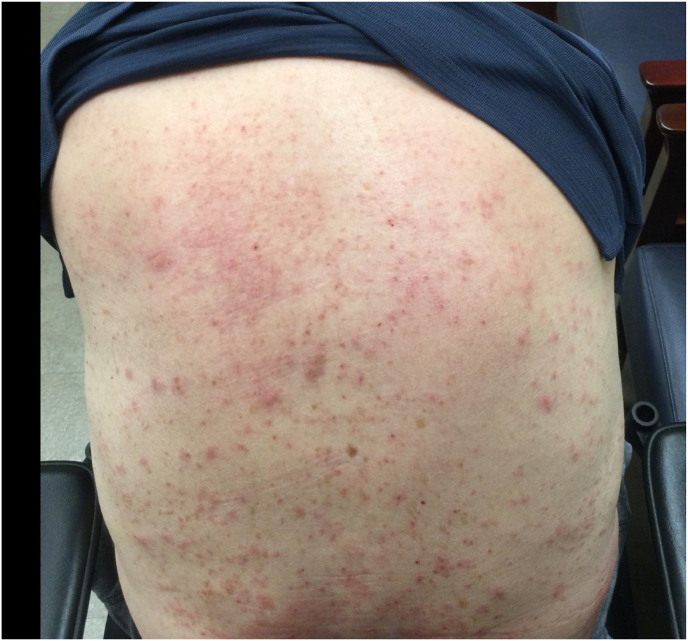
Posterior trunk cutaneous rash on POD 54.

**Fig. 5:**
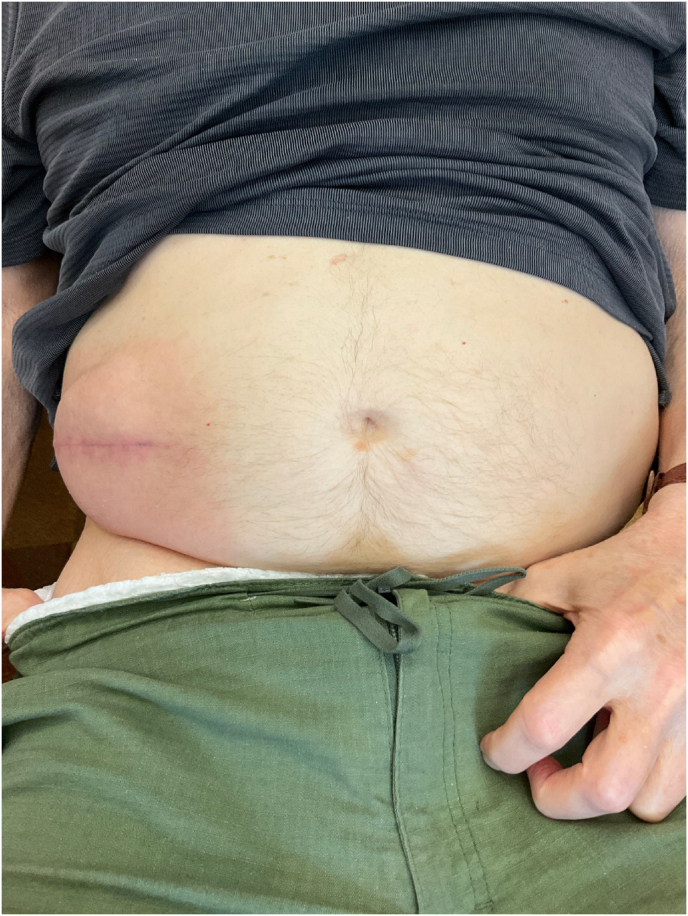
Pump reservoir site incision at POD 64 showing resolution of erythema and skin lesions.

Care should be taken with IDDS patients who develop skin irritation over their pump pocket site to differentiate between localized infection versus other superficial dermatologic skin conditions. A quality history, including questions about the application of topical agents and careful screening for signs or symptoms of infection will guide clinicians towards proper treatment. When dermatologic skin conditions are identified, involving the expertise of dermatology may benefit patients and prevent unnecessary IDDS explanation and therapy interruption.

## Disclosure

C.H. provides general consulting services for Nalu Medical Inc.

## Funding

No funding was received for any portion of the submitted work.

## Declaration of competing interest

The authors declare the following financial interests/personal relationships which may be considered as potential competing interests: Chelsey Hoffman reports a relationship with Nalu Medical Inc that includes: consulting or advisory. If there are other authors, they declare that they have no known competing financial interests or personal relationships that could have appeared to influence the work reported in this paper.

## References

[1] Deer T.R., Pope J.E., Hayek S.M. (2017). The Polyanalgesic Consensus Conference (PACC): recommendations for intrathecal drug delivery: guidance for improving safety and mitigating risks. Neuromodulation.

[2] Deer T.R., Hayek S.M., Grider J.S. (2024). The Polyanalgesic Consensus Conference (PACC)®: intrathecal drug delivery guidance on safety and therapy optimization when treating chronic noncancer pain. Neuromodulation.

[3] Shah N., Di Napoli R., Padalia D. (2025 Jan). StatPearls [internet].

[4] Kinch L., Kohan L. (2020). A case report of reticular telangiectatic erythema following intrathecal drug delivery system reservoir replacement. Neuromodulation.

[5] Zaccarini C.G. (2017). Allergic reaction to an intrathecal baclofen pump leading to pump extrusion: a case report. PM&R.

[6] Bernuz B., Assier H., Bisseriex H., Thiebaut J.B., Rech C., Schnitzler A. (2012). Intrathecal baclofen pump: a foreign-body reaction case report and its solution. J Rehabil Med.

[7] Lundov M.D., Krongaard T., Menné T.L., Johansen J.D. (2011 Dec). Methylisothiazolinone contact allergy: a review. Br J Dermatol.

